# Bio-objects as “boundary crawlers:” the case of microRNAs

**DOI:** 10.3325/cmj.2012.53.285

**Published:** 2012-06

**Authors:** Małgorzata Chrupek, Helena Siipi, Lucia Martinelli

**Affiliations:** 1 University of Rzeszów, Faculty of Biotechnology, Kolbuszowa, Poland chrupek.m@gmail.com; 2 University of Turku, Department of Behavioural Sciences and Philosophy, Turku, Finland helsii@utu.fi; 3 Museo delle scienze, Trento, Italy lucia.martinelli@mtsn.tn.it

## Abstract

**Abstract:**

microRNAs (miRNAs), short RNAs of 21-25 nucleotides, are implied in gene expression and regulation, in biological processes and in human pathologies including cancer. Since miRNAs of plant origin can survive digestion and cooking and enter in animal (including human) sera and tissues, their intervention in mammalian gene expression and regulation might be expected. Mouse experimental feeding, in fact, showed that a miRNA class (MIR168a) is involved in accumulation of the low-density lipoprotein (LDL), the major cholesterol-carrying lipoprotein of human plasma. Considering LDL’s role in atherosclerosis, a negative influence of miRNAs from food origin on our health may be expected. Here we concentrate on the miRNAs’ capability to cross inter-kingdom boundaries through the diet and acting as a “boundary crawler.” The boundary between plant and human is presented under a new perspective, where a new intimate relationship between two genomes – mammalian and plant – belonging to quite different kingdoms is proposed. The food’s role as molecule carrier in our health is also discussed. miRNAs, finally, are presented as an example of “bio-objects” with impact on both medical and cultural issues.

In this essay in a series of articles from “Bio-Objects” research network supported by the Cooperation in Science and Technology (COST) program (1), we present an example of a bio-object: microRNAs (miRNAs). They belong to a nucleic acid category, ie, organic molecules, objects conventionally considered as non-living material, which nevertheless we would here regard as extraordinary candidates to fit into the bio-objects’ group according to some key features we attribute to bio-objects.

miRNAs have been recently detected in eukaryotic cells. They raised the attention of the scientific community and media, following the discovery that miRNAs of plant origin, introduced with the diet, have been found in the sera and tissues of various animals, including humans. Experiments of mouse feeding and an in vitro test that mimics the gastrointestinal tract environment proved that the food-derived plant miRNAs can pass through gastrointestinal tract and enter the circulation and organs. Moreover, food-derived mature plant miRNAs proved to be resistant to cooking and digestion and quite stable in the mammalian serum, blood, and tissues (2).

In this text, the meaning of this discovery will be discussed in the framework of the concept of “bio-object” character and the feature of “boundary crawlers.”

## miRNAs

miRNAs are a family of short RNA organic molecules composed of a number of nucleotides ranging from 21 to 25. First discovered around 20 years ago in the nematode *Caenorhabditis elegans*, many short RNAs were thereafter isolated and nowadays thousands have been identified by random cloning and computational prediction (3). In the early 2000, miRNAs were shown to modulate gene expression at post-transcriptional level by binding to messenger RNA (4). Since then, multiple roles in negative as well as positive regulation of genes have been shown, proving miRNAs’ function in most biological processes such as cell growth, development, and differentiation.

In plants, miRNAs have been found to play key functions in organ development, such as leaf morphogenesis, floral organ identity, and root development. They seem to be involved in various stress responses, such as dehydration, mineral-nutrient, and even mechanical stress (5). Recently, miRNA-based methods of gene silencing have been developed as an important tool for the study of gene function in plants and crop genetic improvement.

In humans, aberrant miRNAs’ expression is related to various diseases such as heart disease, primary muscular disorders, chronic hepatitis, AIDS, polycythemia vera, psoriasis, diabetes, obesity, schizophrenia, fragile-X mental retardation, and Tourette's syndrome, as reported in the Human MicroRNA Disease Database (6). In oncology, miRNAs proved to be down-regulated in breast, lung, and colon cancer, and up-regulated in Burkitt’s and other human B-cell lymphomas (7).

Human miRNAs can be used as highly useful biomarkers, especially for future cancer diagnostics. They rapidly emerge as attractive targets for disease intervention, and miRNA-based therapies are under investigation. Constraints of these therapies seem to be, however, low miRNAs’ in vivo stability since they can be degraded by endogenous RNases, and quick elimination via kidney filtration due to their small molecular mass. Therefore, enhancement of stability and effective delivery strategies are important goals for successful miRNA-mediated gene silencing in medicine. An important step further was a recent discovery that microvesicles can be carriers of circulating miRNAs, which improves miRNA’s stability (2).

Immune-related miRNAs were found in human breast milk and proved to be stable. Accordingly, they might be transferred from the mother’s milk to the infant via the digestive tract, thus playing a critical role in the development of the infant immune system (8).

During research aiming at studying the function of circulating miRNAs in pathology, miRNAs of rice have been surprisingly detected in the serum and plasma of human and animals and the majority of these have been found in microvesicles. miRNA’s origin was easily confirmed with specifics tests (2). Because of their property to act as gene regulators, further research was conducted to clarify whether exogenous diet-derived miRNAs of plant origin accumulated in mammalian blood and tissues were capable to regulate gene expression of mammalians.

It was observed that in mouse, during experimental feeding, the concentration of a specific miRNAclass, MIR168a, varied depending on the quantity of rice dispensed. Moreover, in 31 healthy humans, the presence of nearly 30 types of miRNAs of plant origin was found (2). Finally, sequence database analysis showed the complementarity of MIR168a with nearly 50 mammalian genes, of which the most highly conserved sequence of a putative binding site is located in exon 4 of the “low-density lipoprotein receptor adapter protein 1” (LDLRAP1). It has been found that MIR168a binds to LDLRAP1 and in the liver causes a decreased endocytosis of the low-density lipoprotein (LDL), which subsequently remains in the plasma. LDL is the major cholesterol-carrying lipoprotein of human plasma and it has the crucial role in the pathogenesis of atherosclerosis. Therefore, this result indicates possible negative influence of miRNA from the food on our health (2).

This is just an example of miRNAs’ gene modulation. As different miRNAs can act with different genes it could be possible that they have positive or/and negative effects on our health. So, it might be expected that other miRNAs with possible beneficial effects would be further detected.

## miRNAs as a “boundary crawler”

Concerning human health, agro-food innovation, and possible application in nutrition, we believe that miRNAs may be attributed to the bio-object class. They have, in fact, crucial features of the bio-objects, ie, they are biological products potentially useful for enhancing human life quality and because of their ability to move across different domains they are not stable entities (“boundary crawlers”) (9). They are balancing on the fine line between “natural” and “non-natural”/”artificial.” If evidence of transferring from mother to infant via breast milk would be provided, their capability of crossing individual/individual boundary would also be shown. Moreover their very special feature is the ability to cross plant-mammalian boundaries as here discussed.

### Plant-mammalian boundary

The capability of plant miRNAs to regulate mammalian genes, thus to cross inter-kingdom boundaries, allows to give to these nucleic acids the attribution of “boundary crawlers.” The term “boundary” has normative and non-normative senses. In its non-normative sense the term just refers to something that separates two entities from each other. However, in many contexts, boundary is also something that should not be crossed or crossing of which is somehow extraordinary.

According to our understanding, in academic discussions it is sensible to distinguish between these two senses of the term, and in the context of miRNAs merely to use it in its non-normative sense. The boundary crossings of miRNAs may seem extraordinary for us, but it is not because they are unacceptable or highly uncommon, but because we have just until recently lacked knowledge of them.

miRNAs question the reality of species barrier and existence of species and kingdom boundaries as natural kinds. The concept of natural kinds can be better expressed with the words by Bird and Tobin (10): “To say that a kind is natural is to say that it corresponds to a grouping or ordering that does not depend on humans. We tend to assume that science is successful in revealing these kinds; it is a corollary of scientific realism that when all goes well the classifications and taxonomies employed by science correspond to the real kinds in nature.”

Plant miRNA may enter into our body by food intake, may resist digestion, and may regulate genes. As a result, just through a “natural action” like eating (thus differently from technical interventions such as for instance gene therapy which needs sophisticated equipment), a piece of genetic information from plant origin is delivered into the animal/human organism and is able to regulate our gene expression with the final result to influence our health. Accordingly, we may see the boundary/relationship between plant and human/animal from a new perspective, by perceiving a new intimate relationship between two genomes – mammalian and plant – belonging to quite different kingdoms. It has been already accepted, in fact, that diet may change our health, but it has never been shown in a “direct route.”

### Natural/non-natural

Another nucleic acid group, the xeno-nucleic acids (XNA) are the polymers constructed in laboratory and not found in nature. They are able to store and process genetic information like their “natural” DNA and RNA counterparts (11). We believe it is intuitive perceiving their bio-object quality on the basis of their human technological origin as well as on their ability to balance on the boundary between “natural” and “artificial.”

This is also the case for artificial miRNAs (amiRNAs), which are designed to target one or several genes of interest and provide a new and highly specific approach for effective gene silencing in plants. The miRNAs are a sort of peculiar bio-objects which could be considered as “natural,” as they were discovered in living organisms, but also “artificial” since they have a synthetic counterpart (amiRNA). Thus, miRNAs seem to be natural when they come from food, but non-natural when they are managed via artificial vectors. Additionally, when constructed miRNA (amiRNA) shares all of the properties with another miRNA that has come into being through natural processes, should we not consider the first one as “natural” with respect to its properties and “non-natural” with respect to its origin?

If one accepts the view that naturalness is not merely a question of origin, but may also refer to properties and relations (12), amiRNAs might also be considered “natural” and parts of the living beings.

## Bio-social implications

If further research confirms a role of different quali-quantitative content of miRNAs in the various foodstuffs in our “health-and/or-disease-status,” a new rationale for recognizing the relevance of the diet in our lives will be provided. This fact should enrich these molecules with one of the most relevant features we recognize to bio-objects, ie, the bio-social implication.

Besides availability and control of food resources, in fact, quality and style of diet are relevant topics in our sated society. Incorrect diet habits and food disorders are recognized as notable social problems. Accordingly, nutrition research shifted from epidemiology and physiology to molecular biology and genetics as exemplified by the recent progress of nutrigenomics (13). This latter discipline aims at providing people with methods and tools for disease prevention and health promotion by foods that match the lifestyles, cultures, and genetics, on the basis of personalized dietary recommendation. In addition to nutriogeneomics, so-called functional food (which might in future also contain miRNAs) blurs a distinction between food and medicine.

On the other hand, the notion that to be fit and healthy we need to eat “good” food is a very ancient argument of physicians of both Occidental and Far Eastern medical cultures. Among them, worth remembering are the dictum, “Our food should be our medicine” by Hippokrates of Kos four centuries BCE and the relevance of diet in the classical texts of Chinese traditional medicine starting with Huang Di Nei Jing, two centuries BCE. Cultural, ethical, social, subjective, and psychic implications have also been recognized to food by Herodotus, five centuries BCE, who in The Histories characterized peoples through their food behavior, and by other luminaries, among them Ludwig Andreas Feuerbach (1850) with his famous expression, “der Mensch ist was er isst” (in English “Man is what he eats”). Food that one eats is today often an integral part of one’s identity and lifestyle. Religions and cultures set certain limits to diets of their members, but people also build their identity on lifestyle choices regarding food. One may consider himself as a vegan, member of the slow food movement etc. As already pointed out (14), food choice is a basic form of self-creating, self-expression, and self-definition. With findings regarding miRNAs, this view may be further strengthened to concern not only mental but also the physical self.

In conclusion, being a bio-object on the borders between natural/non-natural and plant/animal, crossing different domains, and carrying bio-social implications, miRNAs can be considered as another example of the impact of life science innovation with potential in both medical and cultural issues.
